# A simple, low-cost, and highly efficient protocol for rapid isolation of pathogenic bacteria from human blood

**DOI:** 10.3389/fmicb.2025.1637776

**Published:** 2025-08-13

**Authors:** Fatma S. Coskun, Joshua Quick, Erdal Toprak

**Affiliations:** ^1^Department of Pharmacology, The University of Texas Southwestern Medical Center, Dallas, TX, United States; ^2^School of Biosciences, University of Birmingham, Birmingham, United Kingdom; ^3^Lyda Hill Department of Bioinformatics, The University of Texas Southwestern Medical Center, Dallas, TX, United States

**Keywords:** rapid diagnostics, bacteria isolation, bloodstream infections, bacteremia, antibiotic susceptibility testing

## Abstract

Bacteremia is a serious clinical condition in which pathogenic bacteria enter the bloodstream, putting patients at risk of septic shock and necessitating aggressive antibiotic treatment. Choosing the most effective antibiotic is crucial not only for resolving the infection but also for minimizing side effects, such as dysbiosis in the healthy microbiome and mitigating the evolution of antibiotic resistance. This requires rapid identification of the pathogen and antibiotic susceptibility testing, yet these processes are inherently slow in standard clinical microbiology labs due to reliance on growth-based assays. Although alternative methods exist, they are rarely adopted in clinical settings because they involve complex protocols and high costs for retraining the personnel and new equipment. Here, we present an optimized and straightforward protocol for the rapid and efficient isolation of bacterial pathogens directly from blood samples, without disrupting standard laboratory workflows. This cost-effective approach utilizes commonly available laboratory equipment and enables direct bacterial cell isolation. By eliminating the need for traditional blood culture steps, it significantly reduces diagnostic delays while remaining fully compatible with downstream bacterial identification analyses. Our protocol achieves over 70% bacteria isolation efficiency within 30 min, remained effective at low bacterial concentrations (1–10 bacteria/0.3 mL blood), and preserved bacterial viability with no notable change in growth lag times. We validated the protocol on several clinically relevant bacterial species, including *Escherichia coli*, *Klebsiella pneumoniae*, and *Staphylococcus aureus*. These findings highlight our protocol’s potential utility in clinical and research settings, facilitating timely cultures and minimizing diagnostic delays. Importantly, the ability to rapidly isolate pathogens may offer critical benefits where timely diagnosis directly influences outcomes. For instance, in a neutropenic cancer patient presenting with fever and signs of sepsis, immediate broad-spectrum antibiotics are typically administered empirically. However, without rapid identification of disease causing pathogens, the risk of inappropriate therapy remains high. By enabling pathogen isolation within 30 min, our protocol can facilitate same-day targeted therapy using molecular or spectrometry-based identification methods, improving early treatment decisions, minimizing exposure to ineffective antibiotics, and potentially reducing ICU admissions and mortality.

## 1 Introduction

Bacteremia is a condition caused by bacteria that translocate into the normally sterile bloodstream, either from external sources or the host microbiome (Adelman et al., 2020). Treating bacteremia is clinically challenging due to uncertainties in identifying the disease causing pathogens, determining its quantity and growth state, and assessing its toxins and antibiotic susceptibility profile. Although many bacteria can grow under standard culture conditions, prior antibiotic treatment may inhibit bacterial growth, rendering identification and susceptibility testing more challenging. Without this information, clinicians must rely on empirical treatments to achieve favorable outcomes. Gram staining, once the pathogen is isolated from a positive blood culture, can quickly distinguish between Gram-positive and Gram-negative organisms and guide narrower-spectrum therapy for increased efficacy (Rand and Tillan, 2006). However, as Gram staining requires a considerable amount of bacteria for imaging, typically from culture-enriched samples, it still introduces a delay before targeted treatment decisions can be made. As a result, patients are often immediately started on multiple broad-spectrum antibiotics to cover both Gram-negative and Gram-positive bacteria. Based on the patient’s response, some drugs may be discontinued while others are added. Although this approach can improve short-term outcomes, it may exacerbate the global antibiotic resistance problem by selecting for resistance genes or resistance conferring genetic changes (Ventola, 2015).

The introduction of antibiotics in the late 1920s has greatly increased life expectancy (Armstrong et al., 1999). However, the effectiveness of antibiotics has been significantly dampened by the rapid emergence of antibiotic resistance which limits their clinical use. According to the Centers for Disease Control and Prevention (CDC), antibiotic-resistant bacteria cause over 2.8 million infections and more than 35,000 deaths annually in the United States alone (CDC, 2019). The solution appears straightforward: clinicians should rapidly identify the bacteria causing the infection and determine their antibiotic susceptibility, allowing for evidence-based treatment regimens that minimize the use of unnecessary or ineffective antibiotics. However, this is a challenging task, as most common clinical microbiology tests are growth-based and therefore inherently slow (Gajic et al., 2022). Furthermore, it is not uncommon for attempts to culture pathogenic bacteria to fail, either due to prior antibiotic exposure or unsuitable laboratory conditions for bacterial growth (Harris et al., 2017).

Currently, the most common method for detecting bacteremia involves plating and incubating blood on special agar plates until the bacterial colonies form (Weinstein and Doern, 2011). A more advanced approach uses diagnostic systems like BACTEC (Washington and Yu, 1971) and BacT/ALERT (Thorpe et al., 1990). The BACTEC system detects bacterial growth by measuring ^14^CO₂ release, which is produced as bacteria metabolize nutrients in the culture medium, making it highly sensitive to even small amounts of bacterial CO₂ production (Weinstein and Doern, 2011). On the other hand, BacT/ALERT uses colorimetric sensors to detect CO₂ level changes, signaling microbial growth with minimal manual handling and reducing contamination risks (Thorpe et al., 1990; Wilson et al., 1995).

Both systems automate the monitoring process, providing real-time alerts to laboratory personnel, thereby helping reduce treatment delays. However, a significant limitation of these methods is their reliance on bacterial growth, which can take several days in some cases. Furthermore, blood cultures marked as positive for bacterial growth after long incubation times do not accurately reflect the true initial bacterial load in the patient’s blood. Moreover, most cells in these samples are still blood cells, making them unsuitable for direct use with most modern sequencing-based detection and phenotyping tools. Therefore, when blood samples test positive in initial screenings, they undergo plating and even sub-plating on differential media such as Durham tubes, MacConkey agar, and/or triple sugar-ferrous sulfate media for identification (Altheide, 2020; Lányi, 1988).

**Figure 1:**
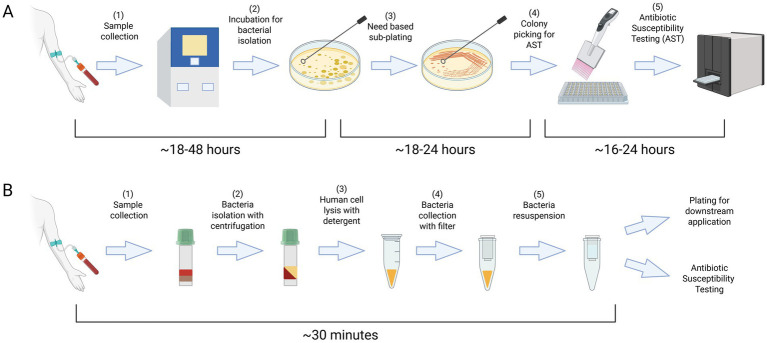
The standard protocol for managing infectious agents typically requires a period of 2–4 days for their isolation and characterization **(A)**. Our enhanced protocol streamlines the bacterial isolation process, reducing the time required to just 30 min with readily available laboratory equipment and consumables **(B)**. Created in BioRender. Coskun, F. (2025) https://BioRender.com/jzd9v5m.

For Gram-positive bacteria, catalase testing is used to differentiate *Staphylococcus* species (catalase-positive) from *Streptococcus* and *Enterococcus* (catalase-negative). For Gram-negative bacteria, culture on MacConkey agar selects for Gram-negative bacilli and differentiates lactose fermenters (e.g., *E. coli*) from non-fermenters (e.g., *Salmonella*). Triple Sugar Iron (TSI) agar provides additional metabolic characterization by detecting glucose, lactose, and sucrose fermentation, gas production, and hydrogen sulfide (H₂S) formation. Durham tube fermentation tests assess gas production from carbohydrate metabolism, aiding in differentiation of organisms such as *E. coli* (gas-positive) and *Shigella* (gas-negative). These classical methods remain valuable for guiding empirical therapy, although they are time-consuming and provide only presumptive identification while awaiting confirmation through automated or molecular tools (Figure 1A).

Once a bacterial isolate is obtained through culture-based methods, more definitive techniques can be employed to achieve species-level identification and assess antibiotic susceptibility with greater precision. Conclusive identification methods include imaging-based platforms and antibiotic susceptibility testing (AST), which are widely used for phenotypic characterization of bacteria (Balouiri et al., 2016; Wikins et al., 1972; Opota et al., 2015; Riedel and Carroll, 2010). Standard antimicrobial testing methods include disk diffusion assays and liquid-based assays, in which bacterial growth is quantified in multiple antibiotics at different doses (Wikins et al., 1972). Traditional methods usually require 1–3 days, but newer approaches speed up the process with varying success. Laser-based technologies like Raman spectroscopy enhanced by deep learning can differentiate bacterial species by analyzing their unique vibrational spectra (Ho et al., 2019). On the other hand, Matrix-Assisted Laser Desorption/Ionization Time-of-Flight Mass Spectrometry (MALDI-TOF MS) can identify microorganisms from pure cultures within minutes by profiling unique spectroscopic signatures (Patel, 2013; Singhal et al., 2015; Croxatto et al., 2012). MALDI-TOF based assays can sometimes be directly employed for positive blood cultures based on bacterial load. In addition to these methods, rapid metagenomics using modalities such as nanopore sequencing and rapid quantitative PCR are emerging as promising alternatives, enabling the detection of bacterial DNA or resistance genes in clinical samples (Zhang et al., 2022; Liu et al., 2012; Quick et al., 2015; Loman et al., 2015). In summary, all emerging techniques for identifying and phenotyping bacteria have shown promise for rapid and precise microbial detection. However, most importantly, efficacies of these advanced techniques often rely on or are significantly enhanced by the availability of pure bacterial cultures achieved by culturing or sub-culturing (Liu et al., 2007; Topic Popovic et al., 2023).

Recent efforts have focused on eliminating the labor-intensive culturing steps to enable direct pathogen identification from blood samples in clinical settings. For instance, the Accelerate Pheno system streamlines this process by combining fluorescence *in situ* hybridization (FISH) with morphokinetic analysis to deliver identification in ~90 min and AST results in ~7 h, significantly faster than traditional methods (Patel et al., 2021; Marschal et al., 2017). It has shown high accuracy (95–99% ID specificity; ~95% AST agreement) and can reduce time to optimal therapy by 18–40 h (Ullberg and Ozenci, 2020). However, it is not a fully culture-independent solution, as it requires a positive blood culture before use. This means the initial culture-based delay still exists. Additionally, its pathogen panel is limited, excluding off-panel and polymicrobial infections (Ullberg and Ozenci, 2020). Its effectiveness also depends on continuous lab staffing and clinician availability, and its specialized instrumentation and consumables pose cost and infrastructure challenges, particularly in resource-limited settings.

PA-100 AST nanofluidic system is another innovative solution for rapid and precise AST testing. Used for urine samples with minimal sample preparation, it won the Antimicrobial Resistance (AMR) Longitude Prize. This system has undergone clinical evaluation, demonstrating its effectiveness in delivering phenotypic antimicrobial susceptibility test results within 45 min (Alonso-Tarres et al., 2024). Meanwhile, emerging technologies such as vacuum filtration, microfluidics-based methods, and flow-based systems remain in developmental stages (Zhao et al., 2019; Yu et al., 2022; Banerjee and Jaiswal, 2018). Despite these recent innovations, their limited availability highlights the urgent need for a simple and practical method to directly isolate bacteria from fresh blood samples for use with diagnostic tools already present in most clinics.

In summary, diagnosing bacteremia in routine clinical practice involves incubating patient blood until bacterial growth is detected, typically through plating or liquid culture, which may take several days (Figure 1A). After isolation, advanced methods like sequencing or imaging can be utilized to identify the bacteria. However, their clinical utility may still be limited as the presence of large number of blood cells interferes with sequencing by masking bacterial genetic signals and hinders imaging by making the localization of bacterial cells difficult. Most of the advanced techniques also depend on isolation of bacterial colonies. Therefore, a simple, robust method to isolate viable bacteria from blood would greatly improve the effectiveness of these diagnostic tools. To address this gap, we developed a simple, rapid, and low-cost protocol that reduces the time needed to obtain bacterial cultures from several days to just 30 min (Figure 1B). In brief, our protocol employs a detergent to selectively lyse host blood cells and uses filter centrifugation to isolate viable bacteria with over 70% efficiency. Using this method, we successfully tested multiple clinically significant bacterial strains, including *Escherichia coli* (*E. coli*), *Klebsiella pneumoniae* (*Kleb*), and *Staphylococcus aureus* (*S. aureus*).

## 2 Materials and equipment

### 2.1 Reagents and materials

- Freshly drawn human blood (K2 Vacutainer, BD367899)
- 1X PBS (Phosphate-buffered saline)
- Filtered 1% saponin solution (Sigma-Aldrich SAE0073)
- Sterile Luria Broth (LB) media and agar plates
- Clinical bacterial strains (e.g., *E. coli*, *K. pneumoniae*, *S. aureus*)

### 2.2 Consumables

- Lithium-heparin separation tubes (BD365985)
- Sterile Eppendorf tubes
- Spin-X centrifuge filters (0.2 μm, Sigma-Aldrich CLS8162)

### 2.3 Equipment

- Tabletop centrifuge (6,000 × g capability)
- Pipettes and sterile filter tips
- Vortex mixer

## 3 Methods

### 3.1 Objective

To develop and validate a protocol that isolates viable bacteria from whole blood within 30 min, maintaining recovery rates >70% even at very low bacterial concentrations.

### 3.2 Bacterial strains

All initial experiments and optimization steps were carried out using a clinical *E. coli* isolate (ATEC) (Rodrigues et al., 2024). Other tested strains include *E. coli* 3,122 B34*, K. pneumoniae* 3,160 B36, and *S. aureus* 3,220 B37. For antibiotic susceptibility and minimum inhibitory concentration (MIC) experiments, ATEC was transformed via electroporation with plasmids encoding either wild-type (WT) or catalytically inactive (DEAD) versions of TEM-1 *β*-lactamase, generating ATEC-TEM-1-WT and ATEC-TEM-1-DEAD, respectively. Electrocompetent ATEC cells were prepared using a rapid plate-based protocol as described by Gonzales et al. (2013), which involves harvesting actively growing bacterial lawns from LB agar, followed by three washes in ice-cold sterile distilled water to generate competent cells. For electroporation, 40 μL of freshly prepared electrocompetent cells were mixed with up to 1 μg of plasmid DNA and transferred into pre-chilled 0.1 cm cuvettes. Electroporation was performed at 1.8 kV, resulting in a time constant of ~5 ms. Immediately after pulsing, cells were recovered in 1 mL LB broth and incubated at 37°C for 30 min before plating onto selective LB agar containing chloramphenicol. For recovery experiments, frozen glycerol stocks of each strain were thawed and spiked into LB broth with or without chloramphenicol (CAM with final concentration of 30 μg/mL) and incubated overnight at 37°C with shaking. Cultures were then streaked onto LB agar plates to isolate single colonies, which were expanded and used for minimum inhibitory concentration (MIC), IC50, and AST analyses.

### 3.3 Protocol overview

*Step 0: preparation of the bacteria*

- Streak bacterial strains from frozen glycerol stocks onto LB agar plates
- Incubate the plate overnight at 37°C incubator
- Inoculate single colony into 5 mL liquid LB
- Culture overnight at 37°C by shaking at 220 rpm

*Step 1: blood spiking and centrifugation*

- Dilute overnight bacterial cultures to OD600 = 1 (~5 × 10^8^ CFU/mL)
- Make serial dilutions in PBS.
- Spike precalculated number of bacteria into 300 μL of blood and mix thoroughly
- Centrifuge at 6000 × g for 2 min in heparin tubes

*Step 2: plasma removal and bacteria collection*

- Remove the plasma (~100 μL) from above the gel barrier in the heparin tubes
- Add 100 μL PBS, pipette gently, and transfer to a sterile tube
- Wash the top portion of the heparin tube with an additional 100 μL PBS and pool the samples in the sterile tube

*Step 3: lysis of blood cells*

- Add 1% saponin, vortex 15 s, incubate at room temperature for ~15 min

*Step 4: bacterial filtration*

- Transfer sample to a Spin-X column, centrifuge at 6000 × g for 2 min
- Wash membrane with 200 μL PBS, vortex, and centrifuge again

*Step 5: bacterial recovery*

- Resuspend retained bacteria in 100 μL PBS by rigorous vortexing
- Repeat this step if necessary to increase recovery yield.
- Plate serial dilutions for CFU quantification and incubate at 37°C or directly start antibiotic susceptibility testing based on the need.

*Pause points:*

- After bacteria collection (Step 2) and filtration (Step 4), samples can be temporarily stored on ice.

*Step 6: MIC determination (optional: 96-well plate assay)*

- A 10-fold serial dilution of ampicillin was prepared across columns 1–10 of a 96-well plate, starting at 100 μg/mL in column 1. Column 11 contained bacterial cells only (no antibiotic) as a growth control, and column 12 contained media only as a background control.
- 300 μL of whole blood was spiked with 20 μL of ATEC-TEM-1-WT or ATEC-TEM-1-DEAD bacterial suspension (OD₆₀₀ = 1.0).
- Bacteria were recovered using our protocol and resuspended in 200 μL of PBS.
- The recovered bacteria were inoculated into 5 mL of LB broth and mixed thoroughly.
- Equal volumes of the recovered culture were immediately added to each well of the ampicillin dilution plate, including the control wells.
- In parallel, an identical plate was inoculated using the spiked-in (pre-recovery) bacteria to assess any differences due to the recovery process.
- Plates were incubated at 37°C for 20 h with shaking at 400 rpm.
- Bacterial growth was measured at OD₆₀₀ using a microplate reader.
- Growth inhibition curves were fitted to a four-parameter logistic regression model.
- MIC was defined as the lowest ampicillin concentration that completely inhibited visible growth (OD₆₀₀ comparable to media-only control).
- IC₅₀ was defined as the ampicillin concentration that reduced bacterial growth by 50%, based on the fitted dose–response curve.

*Step 7: antibiotic susceptibility testing (optional: MicroScan panel).*

MicroScan MIC panel included 29 clinically relevant antibiotics: aztreonam (AZT), cefazolin (CFZ), tigecycline (TGC), amoxicillin-clavulanate (AUG), ampicillin (AM), cefepime (CPE), cefuroxime (CRM), ampicillin-sulbactam (A/S), cefoperazone (CP), levofloxacin (LVX), cefditoren (CFTE), ceftaroline (CFT), amikacin (AK), gentamicin (GM), cefoxitin (CFX), ceftazidime-avibactam (CAZ/CA), ceftaroline-avibactam (CFT/CA), tetracycline (TE), tobramycin (TO), ceftazidime (CAZ), piperacillin (PI), piperacillin-tazobactam (P/T), cefotaxime (CAX), fosfomycin (FD), ticarcillin-clavulanate (TIM), ertapenem (ETP), imipenem (IMP), meropenem (MER), and trimethoprim-sulfamethoxazole (T/S).

- 300 μL of whole blood was spiked with 20 μL of ATEC-TEM-1-WT or ATEC-TEM-1-DEAD bacterial suspension (OD₆₀₀ = 1.0).
- Bacteria were recovered using our protocol and resuspended in 200 μL of phosphate-buffered saline (PBS).
- The bacterial suspension was then inoculated into 20 mL of LB broth.
- 200 μL of the resulting culture was used to inoculate each well of the MicroScan MIC Panel (Beckman Coulter) across all 96 wells.
- As a spiked-in control (pre-recovery reference), 20 μL of the original bacterial suspension was inoculated into 20 mL of LB broth and 200 μL of this culture was also used to inoculate a parallel MicroScan MIC Panel.
- Minimum inhibitory concentrations (MICs) were recorded as the lowest antibiotic concentrations in the panel that completely inhibited visible bacterial growth after 18 h of incubation at 37°C.

## 4 Results

### 4.1 Quantifying bacterial viability with plating

To evaluate our protocol, we spiked known quantities of *E. coli* cells (ATEC strain; see Methods) into freshly collected sterile human blood. This approach was necessary due to the limited availability of patient blood samples from suspected bacteremia cases, especially those not yet treated with antibiotics, and the difficulty of accurately quantifying bacterial load in such clinical samples.

**Figure 2:**
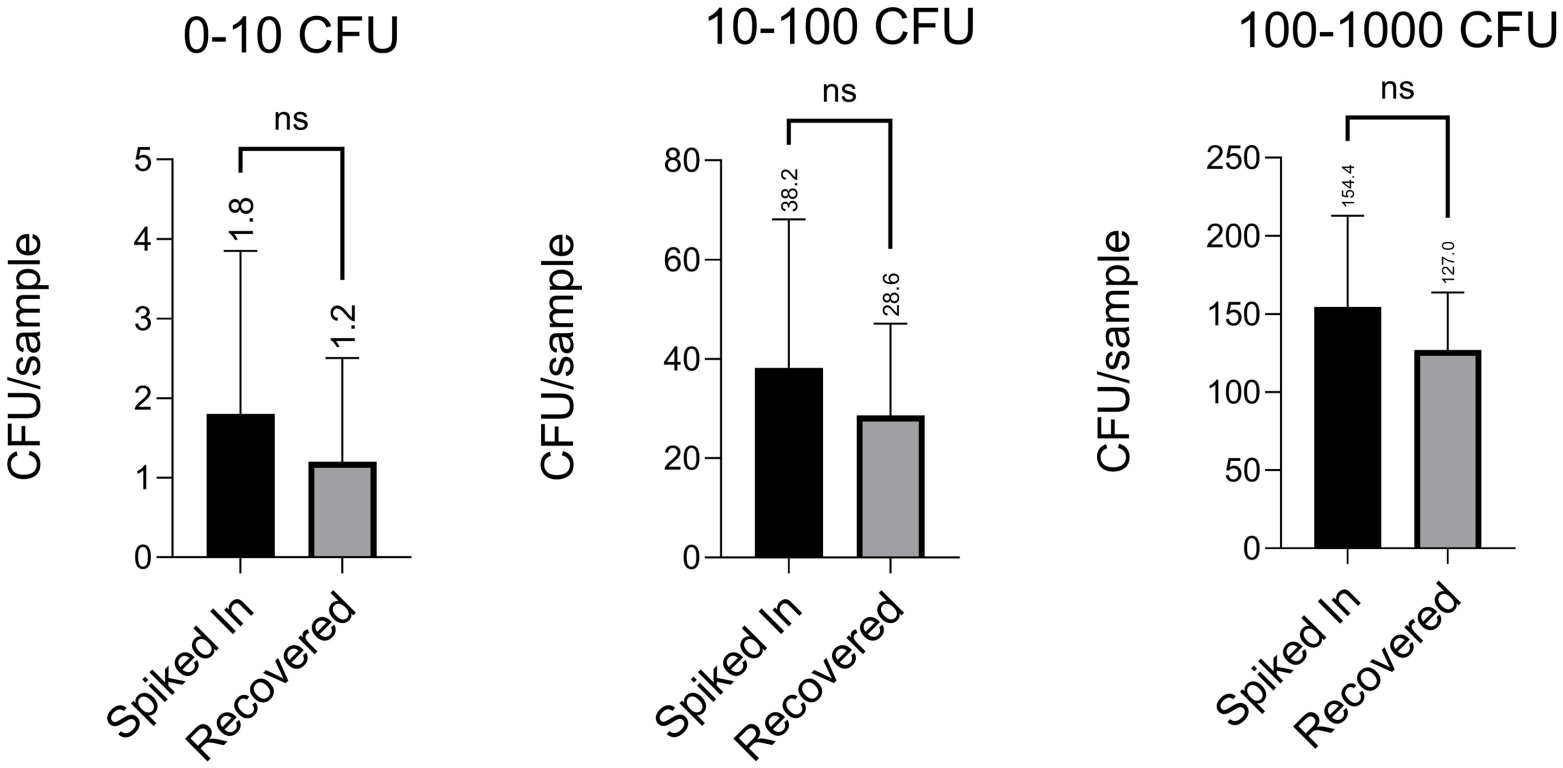
Our protocol achieved recovery rates exceeding 70%, even when tested with extremely low spiked densities of a clinical *E. coli* isolate (ATEC). Each figure displays data from four independent experiments, with five technical replicates per condition in each (i.e., five plates counted per group per experiment). Values are reported as mean ± standard deviation (e.g., 1.80 ± 1.83, 1.20 ± 1.17, 38.20 ± 26.73, 28.60 ± 16.54, 154.40 ± 52.35, 127.00 ± 32.83), and the average of each group is indicated above the corresponding bar. Statistical comparisons were performed using an unpaired two-tailed Student’s *t*-test, unless otherwise noted.

As shown in Figure 2, we tested a range of bacterial cell densities. Blood was drawn from two donors who had not taken antibiotics for at least 2 weeks prior to collection. Freshly drawn blood was spiked with *E. coli*, and bacterial isolation was performed as outlined in the Methods section. Following Step 5, the PBS containing recovered bacteria was plated onto LB agar plates. To monitor for contamination, PBS and 1% saponin alone were plated in parallel during each experiment. The original spiked inocula at respective dilutions were also plated as controls. After overnight incubation at 37°C, colony counts from both recovered and spiked plates confirmed the viability of the isolates.

Across all tested densities, we consistently recovered more than 70% of the spiked bacterial cells. Potential losses were attributed to bacterial adherence to plastic surfaces or cell death during processing. To assess possible losses during wash steps, we also plated the third wash buffers following Step 2 and Step 4; no bacterial growth was observed. Recovery efficiencies across the dilution series ranged from 1.80 ± 1.83 cells added with 1.20 ± 1.17 recovered, to 38.20 ± 26.73 added with 28.60 ± 16.54 recovered, and up to 154.40 ± 52.35 added with 127.00 ± 32.83 recovered, demonstrating consistently high recovery. Notably, even when only one or two cells were added, we recovered the majority, underscoring the robustness of our protocol. In clinical contexts where bacterial loads are often very low, ranging from 1 to 10 CFU/mL^16^, particularly after antibiotic administration, recovering even a few viable cells may be sufficient to initiate downstream diagnostic workflows such as plating, Gram staining, or molecular identification. These results demonstrate that our method performs reliably even under conditions of extreme dilution, where stochastic noise could otherwise hinder accurate recovery.

### 4.2 Quantifying bacterial viability with lag time measurements

**Figure 3:**
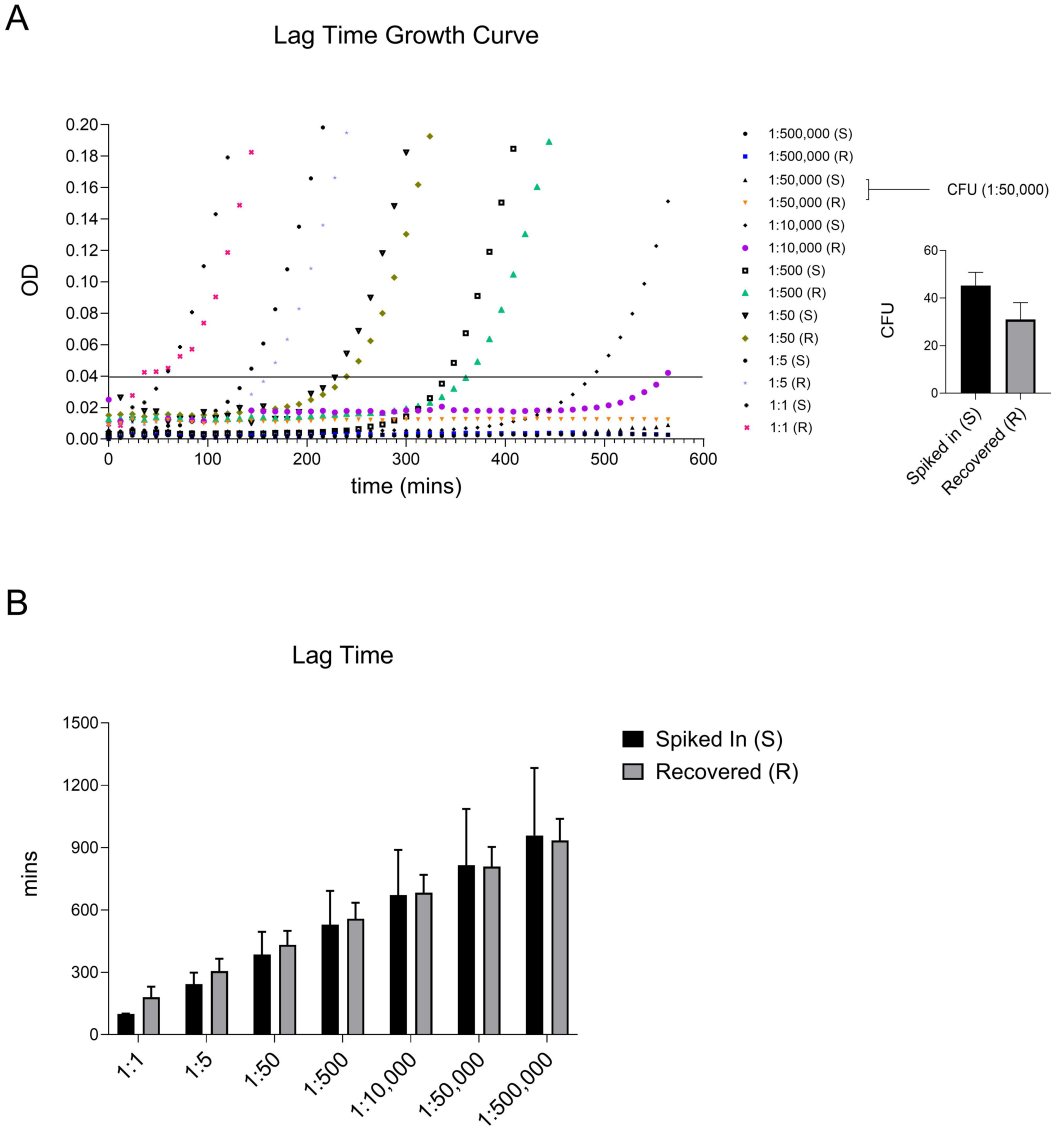
Our protocol did not significantly alter the lag time of recovered ATEC compared to the spiked-in bacteria. Lag time was defined as the time (in minutes) required for the culture to reach an OD of 0.04. A representative growth curve of two independent experiments where ATEC spiked at various dilutions, along with the corresponding CFU counts for the indicated dose, is presented in panel **(A)**. The average lag times calculated from these curves are summarized in panel **(B)** and presented as mean and standard deviation. Statistical comparisons were made using an unpaired two-tailed Student’s *t*-test and showed no significant differences.

A valid concern regarding our bacterial isolation protocol is whether the detergent and washing steps introduce growth delays in recovered bacteria. To address this, we measured the lag times of spiked bacteria and compared them to those recovered from blood. Lag time is typically defined as the duration before bacterial cells enter the exponential growth phase after being diluted to very low densities following overnight growth to saturation (Hall et al., 2014). In our experiments, we defined lag time as the time required for a bacterial culture to reach a background-corrected optical density (OD600) of 0.04, at which standard absorption-based methods are sensitive enough for detection and quantification. At this point, optical density measurements are no longer limited by the limit of detection (LOD) threshold of ~0.005 in our plate reader (Tecan M200PRO). Bacterial cells (100 μL) isolated from blood were transferred to 96-well plates prefilled with 100 μL of 2x concentrated Luria Broth (LB) growth medium. Bacterial growth was monitored continuously by measuring OD600 every 3 min at 37°C, and background-corrected OD600 readings were used to calculate the lag time. For each experiment, the lag time of bacteria not subjected to the isolation protocol was also measured to quantify its effect on bacterial growth delay. A representative growth curve is shown in Figure 3A. In parallel, the number of spiked and recovered viable bacteria was determined by plating cells on LB agar and counting colony-forming units (CFUs) (Figure 3A).

As shown in Figure 3B, there were no significant differences in lag times between the two groups. Notably, high error bars were observed at the lowest tested cell density, likely due to stochastic noise introduced by pipetting variability or uncontrollable factors such as phenotypic heterogeneity among bacterial cells. Apart from this, our protocol did not induce significant delays in bacterial growth. These recovered bacterial cultures can be directly used for identification or antibiotic susceptibility testing, as described previously. However, for sequencing purposes, an additional step involving nuclease treatment and subsequent washing may be required to eliminate host DNA or RNA contamination.

### 4.3 Efficient recovery of bacterial cells from human blood in 30 min

Upon optimizing the protocol with ATEC, we tested its efficacy against different bacterial species. For this, we used clinical strains of Gram-negative bacteria including *Escherichia coli* 3,122 B34 (*E. coli*) and *Klebsiella pneumoniae* 3,160 B36 (*Kleb*), as well as the Gram-positive *Staphylococcus aureus* 3,220 B37 (*S. aureus*). All these species are considered pathogens of concern according to CDC reports (CDC, 2019).

The clinical strains were generously provided by Dr. David Greenberg’s laboratory; with *E. coli* and *Kleb* supplied on MacConkey agar and *S. aureus* on Mannitol Salt Agar. To assess their suitability for downstream experiments, we first evaluated their growth on LB agar plates. All strains were able to grow on LB, although *S. aureus* exhibited slower colony formation, and produced smaller round colonies compared to the Gram-negative isolates. These strains were then spiked into blood and recovered using the same protocol described for the ATEC strain in the Methods section. Colony-forming units were counted the next day for both the spiked-in inoculum and the recovered bacteria to assess recovery efficiency.

**Figure 4:**
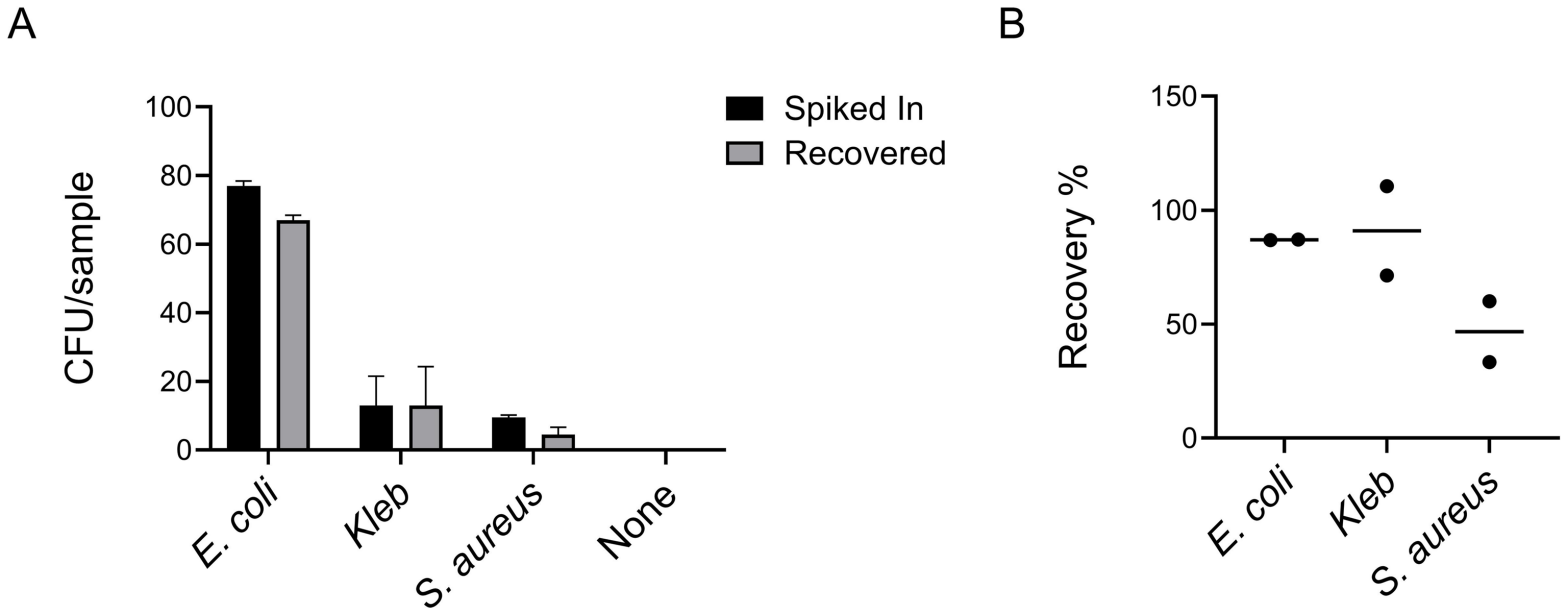
Our protocol demonstrated high efficiency across a set of clinical isolates, encompassing both Gram-positive (*S. aureus*) and Gram-negative (*E. coli* and *Kleb*) bacterial species **(A)**. The graph shown is representative of two independent experiments, each performed with five technical replicates (i.e., five plates counted per condition). Bar graphs display the mean values, with error bars indicating standard deviation. Recovery efficiencies calculated from the averages of the two experiments are expressed as percentages **(B)**.

As shown in Figure 4, our protocol demonstrated high efficiency across a range of pathogens, with recovery rates of approximately 90% for Gram-negative *E. coli* and *Kleb* and around 50% for Gram-positive *S. aureus*. The recovery profiles of the Gram-negative strains, including two *E. coli* isolates and *Kleb*, were comparable and consistently higher than that of the Gram-positive species. The lower recovery efficiency for Gram-positive bacteria is likely due to their distinct morphological characteristics such as being spherical or lacking the outer membrane. Despite variations in growth characteristics and metabolic requirements among these bacteria, the protocol proved highly versatile, preserving bacterial viability effectively for a wide spectrum of pathogens commonly linked to bloodstream infections. Therefore, we expect this protocol to have broad utility in clinical microbiology laboratories.

### 4.4 Colony recovery and ampicillin resistance post-treatment

To determine whether the recovery process leads to plasmid loss, a common cause of resistance loss, we utilized ATEC constructs expressing either functional TEM-1 *β*-lactamase (ATEC-TEM-1-WT) or its catalytically inactive variant (ATEC-TEM-1-DEAD), and assessed their ampicillin resistance in both “Spiked In” and “Recovered” conditions. TEM-1 is a well-characterized class A β-lactamase that hydrolyzes β-lactam antibiotics such as ampicillin, penicillin, and early-generation cephalosporins, rendering them ineffective (Stec et al., 2005; Matagne et al., 1998). It is one of the most prevalent resistance enzymes in *E. coli* and related species (Bajpai et al., 2017). The TEM-1 DEAD construct carries Ser70Ala and Glu166Ala (E166A) mutations, which abolish catalytic activity while preserving expression (Knox et al., 1996). Both versions were expressed from a plasmid carrying a chloramphenicol resistance (CAM) gene, allowing selection.

**Figure 5:**
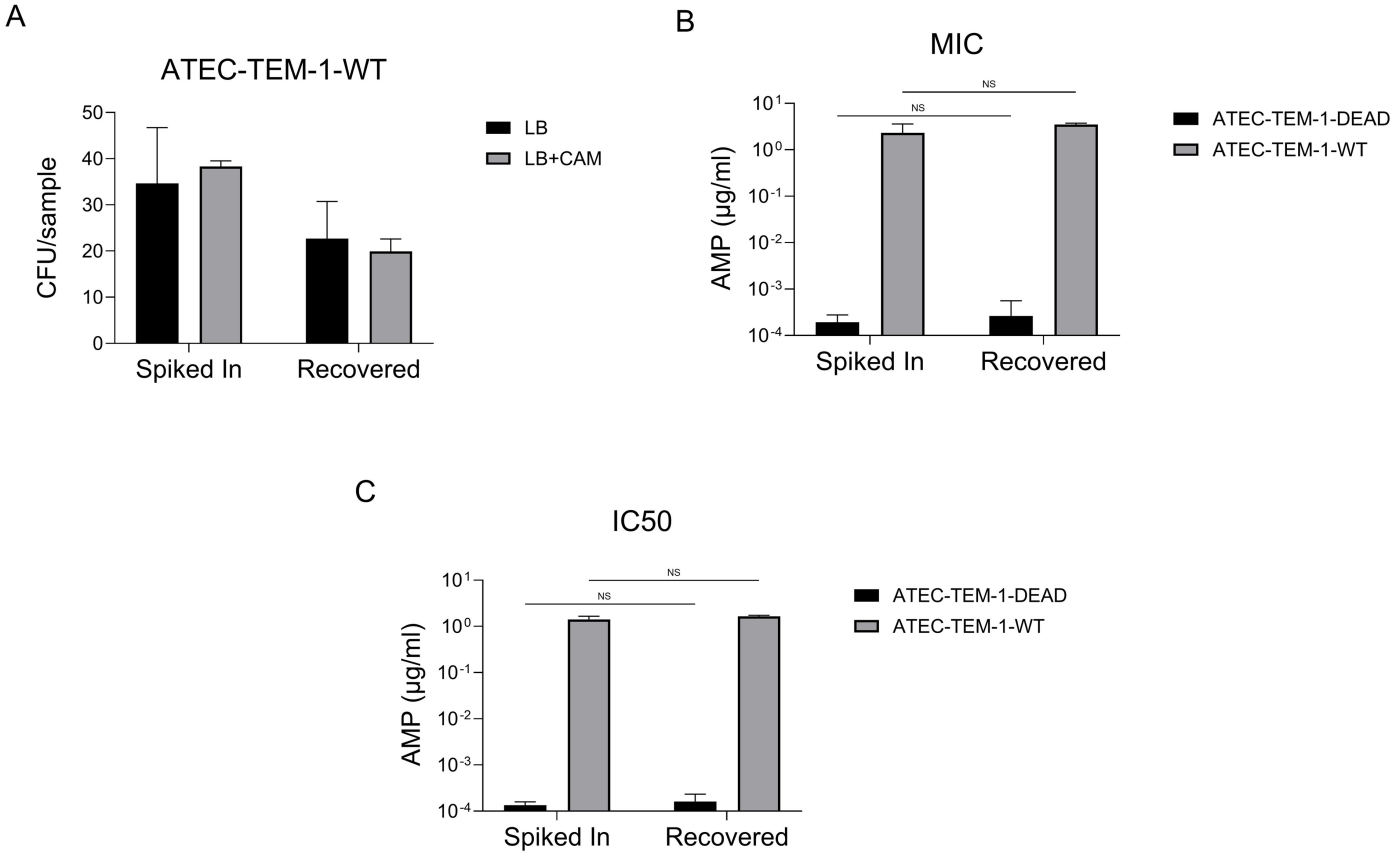
CFU counts for ATEC-TEM-1-WT and ATEC-TEM-1-DEAD strains grown in standard LB and LB supplemented with chloramphenicol (CAM) are shown for both spiked-in and recovered conditions **(A)**. Data represent the average of three replicate plates. The experiment was independently repeated twice with consistent results. MICs and IC₅₀ values for ampicillin were determined using serial dilution assays in microplates **(B)**. IC₅₀ values were calculated from growth inhibition curves fitted to a four-parameter logistic regression model **(C)**. Data are presented on a logarithmic scale for clarity. This experiment was also repeated twice, each in triplicates. Statistical significance was assessed using unpaired *t*-tests, with non-significant differences indicated as “NS.” Error bars represent the standard deviation of the mean.

We quantified CFU per sample in the presence and absence of CAM for both ATEC-TEM-1-WT and ATEC-TEM-1-DEAD strains (Figure 5A). The differences in colony counts between “Spiked In” and “Recovered” samples were consistent with previous observations and remained comparable regardless of CAM selection. These results suggest minimal or no plasmid loss during recovery.

Next, we measured ampicillin susceptibility by determining the minimum inhibitory concentration (MIC) and the IC50, the concentration at which 50% of the population is inhibited, for both WT and DEAD strains (Figures 5B,C). As expected, ATEC-TEM-1-WT exhibited high resistance to ampicillin (~1–10 μg/mL), while ATEC-TEM-1-DEAD remained highly susceptible, with IC50 values in the nanomolar range. Importantly, these resistance profiles were unchanged between Spiked In and Recovered populations, with no statistically significant differences detected (NS by unpaired *t*-test).

Together, these findings confirm that both viability and resistance phenotypes are preserved post-recovery in *E. coli* that carried beta lactam resistance gene, supporting the genetic and phenotypic stability of ATEC constructs during and after selective passaging.

### 4.5 Broad-Spectrum antibiotic susceptibility profiles post-recovery

To assess whether the recovery protocol influences broader antimicrobial susceptibility, we employed a MicroScan MIC panel to evaluate the resistance profiles of ATEC-TEM-1 strains across 29 clinically relevant antibiotics. These included representatives from *β*-lactams, aminoglycosides, fluoroquinolones, tetracyclines, carbapenems, and their combination. Both functional (ATEC-TEM-1-WT) and inactive (ATEC-TEM-1-DEAD) constructs were tested in “Spiked In” and “Recovered” conditions (Table 1).

**Table 1:** MICs for ATEC-TEM-1-WT and ATEC-TEM-1-DEAD strains before and after recovery.

Antibiotic	Spiked In_WT	Recovered_WT	Spiked In_Dead	Recovered_Dead
AZT	<4	<4	<4	<4
CFZ	>16	8	<2	<2
TGC	<2	<2	<2	<2
AUG	>16/8	>16/8	<4/2	<4/2
AM	>16	>16	<8	<8
CPE	<2	<2	<2	<2
CRM	8	8	8	8
A/S	>16/8	>16/8	4/2	4/2
CP	<1	<1	<1	<1
LVX	<0.25	<0.25	<0.25	<0.25
CFTE	<1	<1	<1	<1
CFT	<2	<2	<2	<2
AK	<32	32	<32	32
GM	>8	>8	>8	>8
CFX	<8	<8	<8	<8
CAZ/CA	<0.25/4	<0.25/4	<0.25/4	<0.25/4
CFT/CA	<0.5/4	<0.5/4	<0.5/4	<0.5/4
TE	<4	<4	<4	<4
TO	>8	>8	>8	>8
CAZ	<1	<1	<1	<1
PI	>64	>64	>16	>16
P/T	16	8	<4	<4
CAX	<1	<1	<1	<1
FD	<32	<32	<32	<32
TIM	>64	64	<8	<8
ETP	<0.5	<0.5	<0.5	<0.5
IMP	1	<0.5	<0.5	<0.5
MER	<1	<1	<1	<1
T/S	<2/38	<2/38	<2/38	<2/38

Gray shading indicates entries where a difference was observed.

The ATEC-TEM-1-WT strain retained strong resistance to *β*-lactam antibiotics hydrolyzed by TEM-1, such as ampicillin (AM; >16 μg/mL), ampicillin-sulbactam (A/S; >16/8 μg/mL), and amoxicillin-clavulanate (AUG; >16/8 μg/mL). In contrast, the DEAD variant exhibited markedly reduced MICs for these antibiotics (e.g., AM and A/S at <8 μg/mL and 4/2 μg/mL, respectively), reflecting the absence of catalytic activity. Critically, these susceptibility profiles were preserved post-recovery, with no differences observed between Spiked In and Recovered samples for either construct.

Across other antibiotic classes, all strains, regardless of construct or condition, remained highly susceptible to agents such as cefepime (CPE), ceftazidime (CAZ), cefotaxime (CAX), ceftazidime-avibactam (CAZ/CA), and the carbapenems [ertapenem (ETP), imipenem (IMP), meropenem (MER)], with MICs consistently <1 μg/mL or lower. These data confirm that neither TEM-1 expression nor the recovery procedure compromises susceptibility to advanced-generation cephalosporins or carbapenems.

While minor fluctuations were observed (e.g., CFZ shifting from >16 to 8 μg/mL in WT; PI from >64 to >16 μg/mL in DEAD), such changes remained within a narrow range and were not consistently directional. No systematic increase or decrease in resistance was noted across conditions. For example, P/T (piperacillin-tazobactam) showed a slight decrease from 16 to 8 μg/mL in WT, while AK (amikacin) and GM (gentamicin) values remained stable despite high intrinsic MICs (>32 μg/mL and >8 μg/mL, respectively), possibly reflecting intrinsic properties rather than plasmid-driven effects.

Together, these results underscore the robustness of the ATEC platform: antibiotic susceptibility profiles remain stable before and after recovery, and differences between functional and inactive β-lactamase constructs are clearly preserved. This reinforces the genetic and phenotypic integrity of ATEC strains across clinically relevant drug classes under selective and non-selective conditions.

## 5 Discussion

Antibiotic resistance is a critical public health concern, and any intervention that significantly addresses this challenge and improves health outcomes is highly valuable. Rapid bacterial detection, identification, and antibiotic susceptibility testing are essential for enabling clinicians to make evidence-based decisions when designing antibiotic treatment regimens. Despite numerous advancements in state-of-the-art methods over the past decade, their widespread adoption in clinical settings remains limited. This is largely due to high costs, the need for specialized infrastructure, and the additional burden of retraining clinical microbiology personnel, who are often already overextended. Therefore, there is still a need for simple, cost-effective methodologies that can expedite pathogen detection, identification, and phenotyping without disrupting the workflows of clinical microbiology laboratories.

We developed and optimized a simple protocol for the rapid isolation of bacteria from human blood. This protocol deliberately designed such that it involves only basic centrifugation and pipetting steps, using standard laboratory equipment and commonly available consumables such as lithium-heparin separation tubes, Spin-X centrifuge filters, and a tabletop centrifuge to ensure feasibility in resource-limited settings while integrating smoothly into existing workflows. To evaluate the method’s transferability, we invited personnel from Dr. David Greenberg’s laboratory to blindly test the protocol using only the provided instructions. They reported recovery results consistent with our findings, supporting the ease of implementation. We therefore propose that our method is readily adoptable by clinical laboratory professionals with minimal training.

Although our protocol was developed for the isolation of bacterial pathogens from blood in the context of bacteremia and sepsis, its underlying principles, rapid host cell lysis, bacterial preservation, and filtration-based enrichment, make it broadly applicable to other clinical scenarios where detecting viable bacteria in complex host matrices is essential. One potential area of extension is cerebrospinal fluid (CSF) in suspected meningitis cases, especially when bacterial loads are low or patients have received pre-hospital antibiotics, could benefit from this approach to enable culture or downstream molecular diagnostics. The protocol may also prove useful for joint infections with the analysis of synovial fluid. Moreover, with suitable modifications, such as optimization of lysis buffers and pre-clearing steps, the method could be adapted to isolate fungal pathogens from blood (e.g., *Candida* species in candidemia) or other sterile fluids, where culture is often slow and unreliable.

While our protocol offers a rapid and efficient method for isolating viable bacteria from blood, several limitations should be acknowledged. We recognize that due to the limited availability of blood samples from patients with confirmed bacteremia, our validation experiments were conducted using spiked blood from healthy donors rather than genuine clinical specimens. While this approach enabled a controlled evaluation of recovery efficiency, we recognize that it limits the immediate clinical applicability of our findings, particularly in terms of assessing real-world sensitivity and specificity. We have added a discussion of this limitation and suggest that future work will involve validating the method using blood samples from patients with confirmed bloodstream infections. This will be essential for establishing its diagnostic utility in clinical microbiology laboratories. Although this controlled setup was necessary to assess recovery accuracy across a defined input range, it may not fully capture the complexity of real-world clinical specimens, particularly in the presence of ongoing immune responses or antibiotic exposure.

Our current validation focused on three commonly encountered and relatively robust bloodstream pathogens: *E. coli*, *K. pneumoniae*, and *S. aureus*. We recognize that this may not fully represent the method’s performance with more fastidious or atypical pathogens, which often present greater isolation challenges. Additionally, the impact of prior antibiotic treatment on bacterial recovery was not assessed and remains an important variable for future investigation. Expanding our evaluation to include a broader spectrum of pathogens and clinically relevant conditions will be critical for fully establishing the protocol’s diagnostic utility. Moreover, we observed that the recovery efficiency was notably lower for *Staphylococcus aureus*, a Gram-positive organism, compared to the Gram-negative strains tested. This may reflect intrinsic differences in morphology or membrane structure that influence behavior during isolation. Optimization may be needed for consistent recovery across a broader range of pathogen types, including anaerobes, fastidious organisms, or intracellular bacteria. Although our protocol was successfully transferred to another laboratory with minimal training, broader implementation in diverse clinical settings will require further validation across varying operator skill levels and blood collection conditions. Also, while we demonstrated that the recovered bacteria remain viable and suitable for downstream analysis, we did not directly evaluate compatibility with molecular assays such as PCR, MALDI-TOF, or sequencing. Future studies are needed to assess how well our method integrates with these technologies in a clinical workflow.

By significantly accelerating bacterial isolation, our protocol enables faster access to pure bacterial cultures for downstream diagnostic applications, facilitating evidence-based antibiotic treatment decisions and improving patient outcomes. While expediting isolation, our approach primarily serves as a crucial preparatory step, yielding pure bacterial cultures free from large host cells maintaining high bacterial viability, essential for the effectiveness of advanced detection and phenotyping technologies. Its adaptability further enhances its potential for seamless incorporation into routine clinical workflows. Moreover, modified versions of our protocol could be tailored for the isolation of specific pathogens, such as fungal cells from the bloodstream. Given these benefits, we anticipate broad interest and widespread adoption of our methods among clinical microbiologists.

Importantly, in addition to enabling the rapid recovery of viable bacteria from blood, our protocol maintains critical phenotypic traits, including antimicrobial susceptibility profiles. Using *E. coli* ATEC strains carrying either a functional or catalytically inactive TEM-1 *β*-lactamase, we demonstrated that both colony-forming capacity and ampicillin resistance remained stable following recovery. Resistance measurements, including MIC and IC₅₀ values, were indistinguishable between Spiked In and Recovered samples, indicating minimal plasmid loss or disruption of expression during the recovery process. This is essential, as plasmid instability or selective pressures during isolation could otherwise lead to inaccurate resistance readings, compromising the clinical utility of rapid phenotyping.

Beyond ampicillin, we evaluated susceptibility to 29 clinically relevant antibiotics using a MicroScan MIC panel and found that broad-spectrum resistance profiles were preserved across both ATEC-TEM-1-WT and ATEC-TEM-1-DEAD strains. As expected, the WT strain exhibited high MICs against β-lactam antibiotics that are substrates of TEM-1 (e.g., ampicillin, ampicillin-sulbactam, amoxicillin-clavulanate), while the DEAD variant remained susceptible. These phenotypes were consistently observed in both Spiked In and Recovered groups. Other agents, including ceftazidime-avibactam, ceftaroline-avibactam, cefepime, and carbapenems (ertapenem, imipenem, meropenem), retained low MICs across all conditions, indicating preserved susceptibility to non-substrate classes. Although we observed modest MIC fluctuations, typically 2- to 4-fold, for a few antibiotics (e.g., cefazolin, piperacillin, tobramycin), these changes were neither systematic nor directional and likely reflect biological variation rather than protocol-induced effects.

Collectively, these findings underscore the phenotypic fidelity of our recovery process. The protocol not only preserves viability but also maintains the integrity of resistance phenotypes across a clinically relevant antibiotic spectrum. This stability ensures that susceptibility profiles derived immediately after recovery are representative of the *in vivo* state, allowing for accurate, culture-based diagnostics without distortion from handling artifacts. In clinical settings such as sepsis or bloodstream infections, where rapid therapeutic decisions are essential and molecular diagnostics may miss plasmid-encoded or inducible resistance determinants, our approach offers a practical and powerful tool to link bacterial recovery directly with actionable susceptibility data.

## Data Availability

The raw data supporting the conclusions of this article will be made available by the authors, without undue reservation.
